# Modeling the effects of palm-house proximity on the theoretical risk of Chagas disease transmission in a rural locality of the Orinoco basin, Colombia

**DOI:** 10.1186/s13071-016-1884-8

**Published:** 2016-11-18

**Authors:** Diana Erazo, Juan Cordovez

**Affiliations:** BIOMAC, Universidad de los Andes, Carrera 1 E No. 19A 40, Bogotá, 111711 Colombia

**Keywords:** Mathematical modeling, Chagas disease, *Rhodnius prolixus*, Risk factors, House infestation, Palm proximity

## Abstract

**Background:**

Chagas disease is a major public health concern in Latin America and it is transmitted by insects of the subfamily Triatominae, including *Rhodnius* spp. Since palm trees are ubiquitous in Colombia and a habitat for *Rhodnius* spp., the presence of palms near villages could increase contact rates between vectors and humans. Therefore, knowing whether a relationship exists between the proximity of palms to villages and the abundance and distribution of vectors therein, may be critical for Chagas disease prevention programs. Adapting a mathematical model for *R. prolixus* population dynamics in a small village, we model the implications of changing distances between palms and dwellings, to the risk of Chagas disease infection.

**Methods:**

We implemented a mathematical model that reflects *R. prolixus* population dynamics in a small village located in the department of Casanare (Colombia) to study the role of palm-house proximity. We varied the distance between palms and houses by monitoring the network global efficiency metric. We constructed 1,000 hypothetical villages varying distances and each one was run 100 times.

**Results:**

According to the model, as palm-house proximity increases, houses were more likely to be visited by triatomine bugs. The number of bugs per unit time increased progressively in a non-linear fashion with high variability. We stress the importance of village configuration on the model output.

**Conclusions:**

From a theoretical perspective, palm-house proximity may have a positive effect on the incidence of Chagas disease. The model predicts a 1% increase in new human cases per year when houses and palms are brought closer by 75%.

**Electronic supplementary material:**

The online version of this article (doi:10.1186/s13071-016-1884-8) contains supplementary material, which is available to authorized users.

## Background

Chagas disease is a major vector-borne disease in Latin America, caused by the parasite *Trypanosoma cruzi*. The parasite is mainly transmitted to mammal hosts by the feces of triatominae blood-sucking insects [1]. Since palm trees are one of the most common habitats of sylvatic triatomines and these trees often are near villages, palms constitute a risk factor for human infection [2–7].

Recent studies have reported that about 38 species of large palm trees with complex crowns are often infested by triatomines [8]. While some triatomines are opportunistic palm inhabitants, one study suggested that most of the species in the tribe Rhodniini are palm-specialized [9]. In Colombia, natural infections with *T. cruzi* have been found in 15 triatomine species [10], with *Rhodnius prolixus* as the main vector since it has domiciliary anthropophilic habits and has a wide geographical distribution in the Orinoco region [7, 11, 12].

Previous work from our group in the municipality of Mani Casanare (Orinoco region) [12], found that *Attalea butyracea*, a large-crowned palm, is ubiquitous and has large *R. prolixus* densities [7, 13] with high natural rates of infection with *T. cruzi* [12]. Interestingly, the same study reported low colonization indices of households [12] and residents mentioned often seeing adult insects come by at night supposedly attracted by lights [14]. Taken together, the close vector-palm association and the proximity between houses and palms, despite low domiciliation, create a unique mixture of risk factors that may partially explain the endemicity of the region [7].

The quantitative relationship between multiple factors that impact insect migrations from palms trees (e.g., light and distance between sylvatic and domestic habitats) and the risk of human infections is minimally understood [14–16]. In this study, we focus solely on the relationship between the proximity of palms to village dwellings and the possible risk of Chagas disease infection among the village inhabitants.

## Methods

As described in Additional file 1 and [14], we developed a mathematical model that studies *R. prolixus* population dynamics in a village, which includes movement between habitats or patches (i.e. houses and palm trees).

In this study, our model assumes that insect movement between patches is determined by two interacting mechanisms: (i) proximity between patches promotes insect migration; and (ii) light makes a house twice as attractive for an insect compared to the same house without a light. The assumed mathematical relation between distance, light presence and habitat attractiveness is presented in Additional file 1. In this study we wanted to artificially alter the average distance between houses and palms while maintaining a constant light stimuli in the village (to test only for the effects of distance). To do so, we maintained the palms at their original occurrence site and changed the location of the houses.

To compute average distance between houses and palms, we use a modified version of the network metric, *global efficiency* (GE), from Latora & Marchiouri [17]. The concept of network *efficiency* indicates how easily information travels between network nodes. In our study, network *efficiency* measures the ease for insects to move between two types of patches, i.e. dwellings and palms. GE is a measure of functional integration and computed as the inverse of the common Average Path Length (APL) [18]. Unlike APL, GE can be computed on a disconnected network because movements between detached patches are defined to have infinite APL, which corresponds to zero GE [19]. Since our focus was palm proximity, we computed the GE using only the connections that have palms as the origin patch and houses as the destination patch. We called this measure the palm-house efficiency (PHE).

We created 1,000 scenarios in which palm locations were fixed but house locations changed randomly while remaining within the village-limit. For each scenario, we computed the PHE and found that it naturally varied between 0.1 and 1. In our study, we were not able to randomly generate PHE values below 0.1. Note that if we artificially arrange house locations corresponding to a zero PHE, the network between houses and palms is entirely disconnected. A PHE equal to 0.1 produced an average of 2.65 connections, with an average distance of 228 m between houses and palms (out of the 3030 possible connections). For a PHE equal to 1, we obtained 63.24 connections with an average distance of 194 m.

During each scenario run, we computed two epidemiological indices: the proportion of visited houses at steady state (PVH) and the average number of insects per visited house per unit of time (VI). In addition, we computed the number of clusters (NC) and the proportion of patches in the biggest cluster (PPBC).

## Results and discussion

**Fig. 1:**
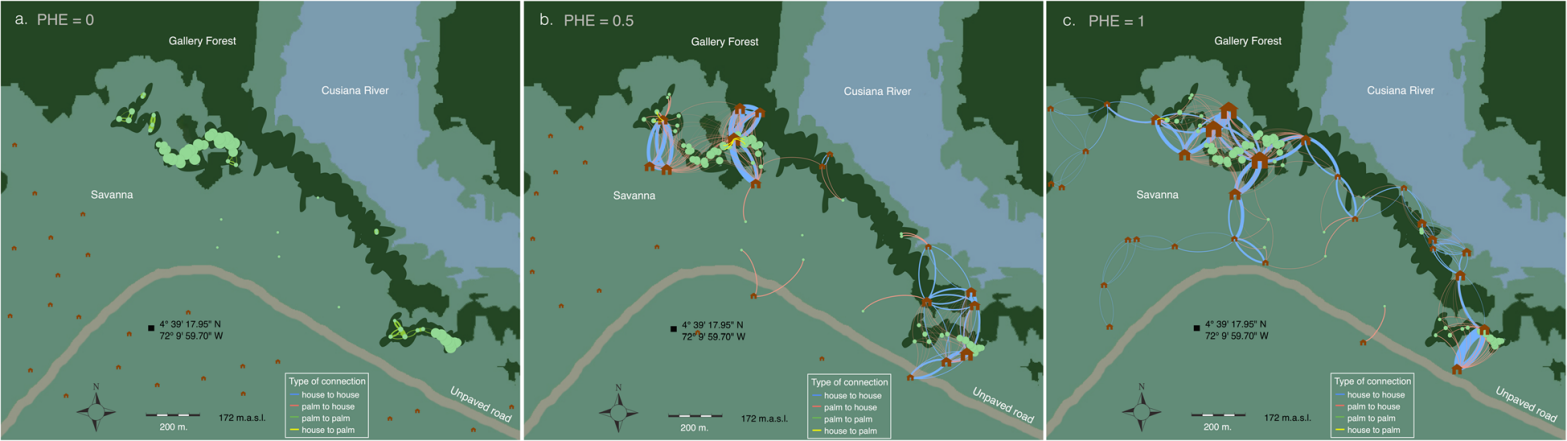
Networks panels. For illustration purposes, we show a network example of the lowest (**a**), medium (**b**) and highest output (**c**) of Palm-House Efficiency (PHE) at steady state. Village landscape was maintained including palm locations. Locations of human dwellings were changed among scenarios to vary PHE. Circle and house icons symbolize palm trees and human dwellings, respectively, and the number of individuals visiting a habitat is represented by the icon’s size. The color of the connections between habitats illustrates the direction of the link (house to house: *blue*; palm to house: *red*; house to palm: *yellow*; and palm to palm: *green*) and their width is related to the flow of insect moving from an origin to a destiny patch. When the PHE is equal to zero (**a**) there are no connections between houses and palms. As PHE increases (**b**) more connections appear because houses with lights at reachable distances increase in the village. Panel **c** shows a simulation in which a high proportion of human dwellings are visited due to an increase in the overall network connectivity

All the runs started with the same initial conditions: palms at their carrying capacity and no individuals in houses. Initial conditions that assumed that not every palm is infested produced very similar results so we decided to go with the worst-case scenario. Figure 1 shows the village configuration network for low, intermediate and high PHE, panels a to c respectively, obtained after a single model simulation.

**Fig. 2:**
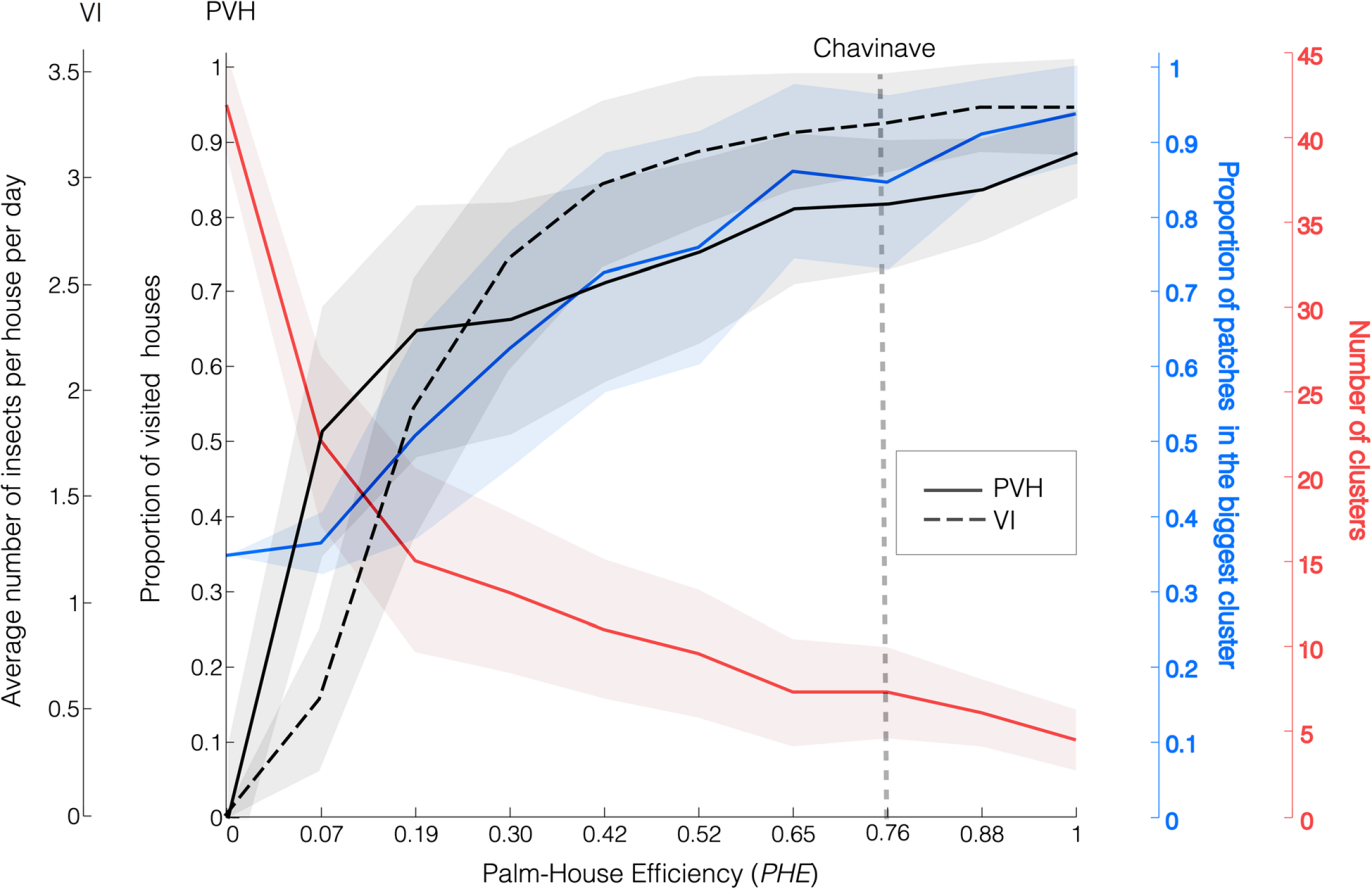
Epidemiological indices. Palm-House Efficiency (PHE) as a proxy for palm-house proximity an its relation with the proportion of visited houses (PVH) and the average number of insects visiting a house (VI). Additionally, we include two relevant network metrics: the number of clusters (NC: *red*) and the proportion of patches in the biggest cluster (PPBC: *blue*). The shadows of the output reflect the standard deviation among simulations for each PHE. PVH and VI show a saturation effect; however a PPBC increments linearly as PHE increases. Our study village has a PHE of 0.76 with an average of 3 individuals per day per house and 93% of the houses visited by triatomine bugs

Our results showed that a higher PHE (i.e. a close proximity between palms and houses) resulted in an increase of the epidemiological indices (Fig. 2). When PHE is equal to 0.01, PVH and VI are low: less than 5% of the houses are visited and have less than one visiting insect per house per day (if PHE is zero then PVH and VI are zero). Increasing PHE to 20% produces a PVH increase of about 60%, while VI increases to 2. This increase in PHE is accompanied by a notable decline in the NC from 39 to 16 clusters, and a 15% increase in PPBC that reflects habitat integration. From this point, as we continue to increase PHE, the mean and standard deviation of PVH and VI follow a linear trend, reaching a PVH equal to 0.9 and VI equal to 3.2.

**Fig. 3:**
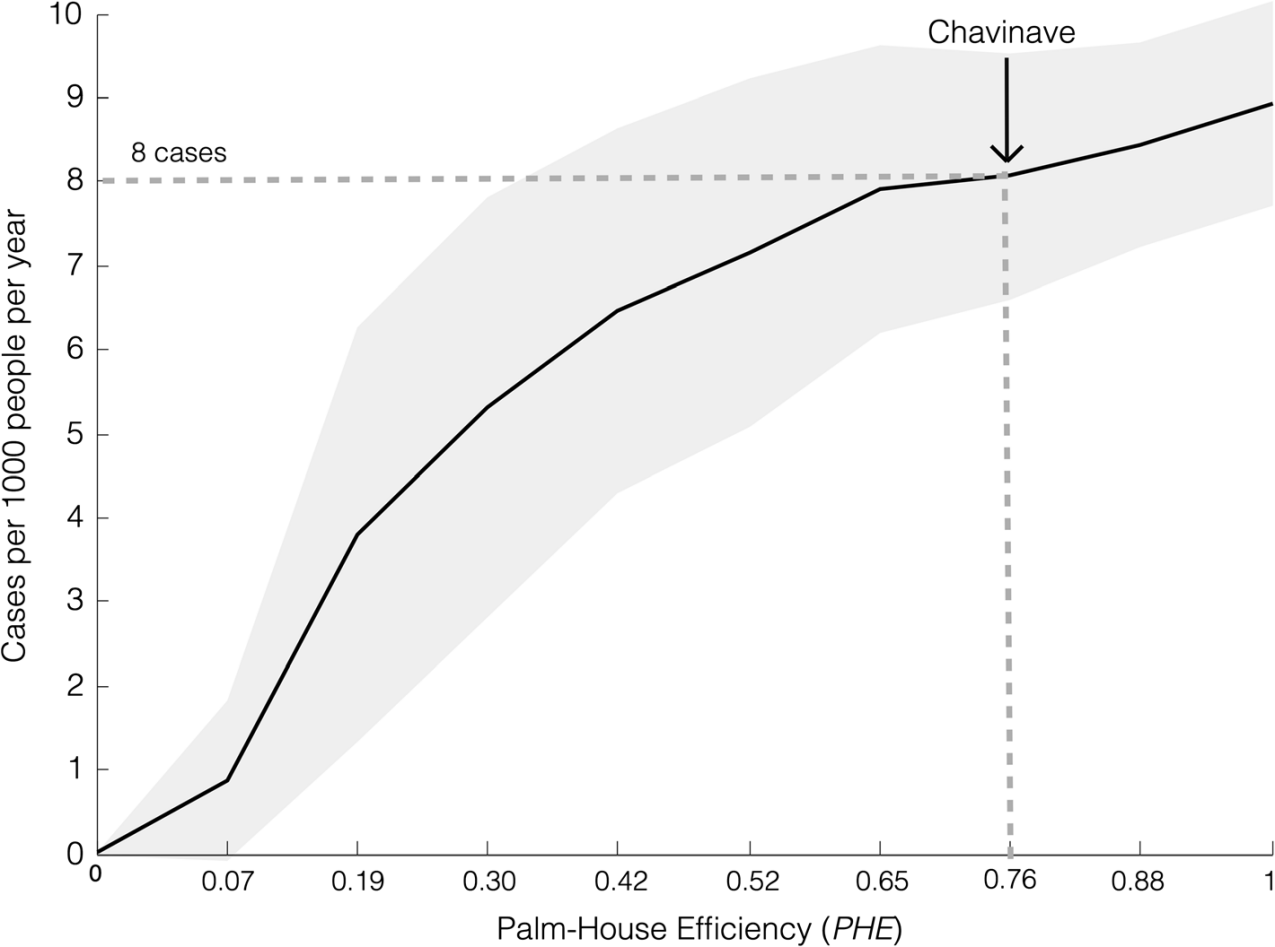
Predicted number of cases as function of PHE. We computed the number of new cases per human per year based on the model output indices: VI and PVH. Additionally for this calculation, we considered reported values such as proportion of infected insects, insect biting rate, triatomine feeding rate on humans and probability of transmission per contact with an infected triatomine. For our study area, Chavinave village, the model predicted 8 cases per 1,000 people per year. For further details, see Additional file 1

Using VI and PVH, we estimated the effect of palm proximity on the risk of Chagas disease infection. To this end, we used *R. prolixus* natural infection in the region, the estimated triatomine bug feeding rate on humans, and the probability of transmission per contact with an infected triatomine (for further details see the appendix and reference [14]). We found that the number of per-capita cases per year has a saturation effect. When PHE is equal to 7%, we computed an average of 1 case per 1,000 people per year, reaching an average maximum of 8.9 cases. However, higher variability is observed at intermediate values (Fig. 3) suggesting that the same PHE could result in different epidemiological situations. Chavinave has a PHE of 76% and a population of 122 people. Thus, using the model we estimate a new case every year. If we could hypothetically alter PHE to be 30%, by relocating dwellings, then the number of cases could be reduced by 50%.

A recent study suggested that at low PHE human risk infection due to vectorial transmission would be very low [20]. When PHE increases, high variability of epidemiological variables occurs among various studies and the need to examine particular house configurations within villages is critical [21–23]. In practice, the level of house-palm proximity can vary by at least three simple mechanisms: (i) keeping a single palm or a few palms near houses in the middle of the village [8]; (ii) introducing a single house or few houses into or near a gallery forest [21]; and (iii) adding a connected network of dwellings to a house that is connected to the forest. These scenarios result in high PPBC and low NC. These types of networks allow for the movement of sylvatic triatomines first from palms to a single house, and then to the rest of the village.

Under some simulations, we observed the formation, merely by chance, of a palm-only network detached from human dwellings. This network was capable of sustaining insect populations that were confined to the sylvatic cycle. This situation may be supported biologically if insects have other feeding sources, such as sylvatic vertebrates possibly associated with palms that could eliminate their need to feed on humans [6, 24].

## Conclusions

In conclusion, our model predicts that palm-house proximity could play a significant role in human infection risk with *T. cruzi*, as suggested by Feliciangeli et al. [20]. Moreover, Saldaña et al. [25] reported dogs in a rural location in Panama, were 11.6 times more likely to become infected if *Attalea butyracea* palms were present in the peridomicile. The effectiveness of vector control strategies could increase by considering the flying ability of triatomine bugs [15] and recognizing the role that palm-house proximity plays in house infestation and re-infestation after control strategies [23, 26]. For instance, one could control vectors using efficient physical barriers, such as window screens, on targeted dwellings near palms [26–28]. As a control strategy, we may be able to reduce insect migration from palms by reconsidering the location of dwellings near palms and creating a buffer zone surrounding such dwellings.

## Additional file

Additional file 1: Mathematical model description, parameters and *Trypanosoma cruzi* infection prediction. (DOCX 102 kb)

## Abbreviations

APL: Average path length
GE: Global efficiency
NC: Number of clusters
PHE: Palm-house efficiency
PPBC: Proportion of nodes in the biggest cluster
PVH: Proportion of visited houses
VI: Visiting index
