# Supplementary material for: Modeling the effects of palm-house proximity on the theoretical risk of Chagas disease transmission in a rural locality of the Orinoco basin, Colombia
Source: Parasit Vectors. 2016 Nov 18;9:592. doi: 10.1186/s13071-016-1884-8 (PMC5116207; doi:10.1186/s13071-016-1884-8)
Supplement: Additional file 1: — Mathematical model description, parameters and Trypanosoma cruzi infection prediction. (DOCX 102 kb) [file 13071_2016_1884_MOESM1_ESM.docx]

**Additional file 1**

*Mathematical Model*

The model accounts for *Rhodnius prolixus* meta-population dynamics in a village at a patch level. For Chavinave village, a patch *i* could be a house or a palm where we consider an age-structured population. $E_{i}(t)$, $N_{i}(t)$ and $A_{i}(t)$ denote the number of eggs, nymphs and adult respectively at time . Additionally, we included migration of adults between patches. The system of ordinary differential equations of the proposed model are:

$\frac{dE_{i}}{dt}=\lambda A_{i}-\frac{1}{\tau}E_{i}-\delta_{E}E_{i}$ (1)

$\frac{dN_{i}}{dt}=\frac{1}{\tau}E_{i}\left(1-\frac{N_{i}+A_{i}}{K_{i}}\right)-\frac{1}{\gamma}N_{i}-\delta_{N}N_{i}$ (2)

$\frac{dA_{i}}{dt}=\frac{1}{\gamma}N_{i}-\delta_{A}A_{i}-\beta_{i}A_{i}+\sum_{j=1}^{n} \alpha_{ji}\beta_{j}A_{j}$ (3)

where $\lambda$ is the per-female egg number, $\tau$is the average hatching time, $1/\gamma$the average nymph to adult maturity rate, $K$is a patch’s carrying capacity and, $\delta_{E}$, $\delta_{N}$ and $\delta_{A}$are egg, nymph and adult per-capita mortality, respectively.

The last two terms of the rate of change of $A_{i}(t)$ refer to adult emigration from and immigration to patch *i*, where $\beta_{i}$ is defined as a per-capita migration and $\alpha_{ji}$ is the adjacency matrix of the network composed by all the patches. $\beta_{i}$ is assumed to have saturation kinetics described by the following first order Hill-equation:

$\beta_{i}=\frac{\sigma(N_{i}+A_{i})}{\eta+(N_{i}+A_{i})}$ (4)

where $\sigma$ is the maximum per-capita migration rate and $\eta$ is the number of individuals at which half of the maximum per capita migration rate occurs. This sigmoid curve will produce a realistic effect: at low densities a few insects will be motivated to move but as the population starts to increase adult migration increases, reaching a maximum rate. The maximum rate was chosen such that it reflects a biological maximum. For example, a palm-house dyad with the palm hosting 20 insects (10 nymphs and 10 adults - carrying capacity) will produce a flow of 1 adult insect per day (10%) to the house at the maximum rate (after 10 days, at his rate, the palm will be adult empty). Obviously, as soon as the abundance starts to decrease the flow will diminish proportionally to the density returning to a lower level. Note that if there are no adults present the flow will be zero despite the number of nymphs.

The entries on the adjacency matrix $\alpha_{ji}$ determines the target habitat *i* from habitat *j* and every entry is defined by:

$\alpha_{ji}=(L_{ji}+1)(200-D_{ji})$ $if$ $D_{ji}>200$ $then$ $\alpha_{ji}=0$ (5)

where $D_{ji}$is the Euclidean distance between patch $j$ and patch $i$ *,* and $L_{ji}$ is a matrix with 1 at entry $j,i$ if patch $i$ has a light that can be seen from patch $j$ and 0 otherwise*.* $\alpha_{ji}$ assumes that the maximum distance an insect can overcome is 200 mts [1].

The parameters included in the mathematical model are described in the following table:

| **Symbol** | **Name** | **Units** | **Value** | **Ref** |
| --- | --- | --- | --- | --- |
| $\lambda$ | Birth rate | $\frac{individual}{day\cdot individual}$ | 1.3 | [2] |
| $\delta_{E}$ | Egg mortality rate | $1/day$ | 0.001 | [2] |
| $\delta_{N}$ | Nymph mortality rate | $1/day$ | 0.004 | [3] |
| $\delta_{A}$ | Adult mortality rate | $1/day$ | Palms: 0.005  Houses: 0.05 | [3]  - |
| $\tau$ | Residency time from egg to nymph | $day$ | 15.4 | [2] |
| $\gamma$ | Residency time from nymph to adult | $day$ | 211 | [2] |
| $K_{i}$ | Carrying capacity in patch $i$ | $individual$ | Palms: 20  Houses: 1 × 10^-3^ | [4–6] |
| $\sigma$ | Maximum per capita migration rate | $\frac{individual}{day\cdot individual}$ | 0.1 | - |
| $\eta$ | Number of individuals at which half of the maximum per capita migration rate occurs | $individual$ | 1 × 10^-6^ | - |
| $L_{ji}$ | Presence of light at destiny patch $i$ seen from patch $j$ | *arbitrary units* | 0,1 | - |
| $D_{ji}$ | Euclidean distance between patch$j$ and patch $i$ | $meter$ | - | - |
|  | Maximum dispersal distance *R. prolixus* | $meter$ | 200 | [1, 7] |

*Trypanosoma cruzi infection prediction*

To estimate the number of cases with *T. cruzi* infection in humans as a function of palm proximity we used the model output indices VI and PIH along with the following reports: *R. prolixus* natural infection in the study area (60.3% [5]), Triatomine bug feeding rate (once every 3–6 weeks [8]), *R. prolixus* feeding frequency on humans (0.583 [9]) and probability of transmission per contact with an infected Triatomine (5.8 × 10^-4^ [10]). Therefore, we obtain 3.9 × 10^-4^ new infected people per insect per year. For Chavinave village with a human population of 122 people (2012), an average of 3.25 insects per house per year and 85% of infested houses would result in 1 new case every year.

**References**

1. Schofield CJ, Matthews JN. Theoretical approach to active dispersal and colonization of houses by *Triatoma infestans*. J Trop Med Hyg. 1985;88:211–22.

2. Arévalo A, Carranza JC, Guhl F, Clavijo JA, Vallejo GA. Comparación del ciclo de vida de *Rhodnius colombiensis* Moreno , Jurberg & Galvão , 1999 y *Rhodnius prolixus* Stal , 1872 (Hemiptera, Reduviidae, Triatominae) en condiciones de laboratorio. Biomédica*.* 2007;27:119–129.

3. Chaves LF, Hernandez M-J, Revilla T a., Rodríguez DJ, Rabinovich JE. Mortality profiles of *Rhodnius prolixus* (Heteroptera: Reduviidae), vector of Chagas disease. Acta Trop*.* 2004;92:119–125.

4. Angulo VM, Esteban L, Luna KP. *Attalea butyracea* próximas a las viviendas como posible fuente de infestación domiciliaria por *Rhodnius prolixus* (Hemiptera: Reduviidae) en los Llanos Orientales de Colombia. Biomédica. 2012;32:277–285.

5. Rendón LM, Guhl F, Cordovez JM, Erazo D. New scenarios of *Trypanosoma cruzi* transmission in the Orinoco region of Colombia. Memórias do Inst Oswaldo Cruz. 2015; volume:110(3):283-288.

6. Jácome-Pinilla D, Hincapie-Peñaloza E, Ortiz MI, Ramírez JD, Guhl F, Molina J. Risks associated with dispersive nocturnal flights of sylvatic Triatominae to artificial lights in a model house in the northeastern plains of Colombia. Parasit Vectors*.* 2015;8:600.

7. Zeledón R, Rabinovich JE. Chagas’ disease: an ecological appraisal with special emphasis on its insect vectors. Annu Rev Entomol. 1981;26:101–33.

8. Rabinovich J, Schweigmann N, Yohai V, Wisnivesky-Colli C. Probability of *Trypanosoma cruzi* transmission by *Triatoma infestans* (Hemiptera: Reduviidae) to the opossum *Didelphis albiventris* (Marsupialia: Didelphidae). Am J Trop Med Hyg. 2001;65:125–130.

9. Feliciangeli MD, Carrasco H, Patterson JS, Suarez B, Martínez C, Medina M. Mixed domestic infestation by *Rhodnius prolixus* Stal, 1859 and *Panstrongylus geniculatus* Latreille, 1811, vector incrimination, and seroprevalence for *Trypanosoma cruzi* among inhabitants in El Guamito, Lara State, Venezuela. Am J Trop Med Hyg. 2004;71:501–5.

10. Nouvellet P, Dumonteil E, Gourbière S. The improbable transmission of *Trypanosoma cruzi* to human: The missing link in the dynamics and control of Chagas disease. PLoS Negl Trop Dis. 2013;7:e2505.
